# The Food and Agricultural Organization and the institutionalization of fishery biology in Brazil, 1955-1978: complexities of the circulation of scientific knowledge

**DOI:** 10.1590/S0104-59702024000100026

**Published:** 2024-06-17

**Authors:** Adriana Feld, Ezequiel Sosiuk

**Affiliations:** i Investigadora adjunta, Consejo Nacional de Investigaciones Científicas y Técnicas. Buenos Aires – CAB – Argentina feldri75@yahoo.com.mx; ii Becario posdoctoral, Consejo Nacional de Investigaciones Científicas y Técnicas. Buenos Aires – CAB – Argentina sosiuk_gm@hotmail.com

**Keywords:** Brazil, Food and Agriculture Organization (FAO), Fishery biology, Expert knowledge, Brasil, Food and Agriculture Organization (FAO), Biología pesquera, Conocimiento experto

## Abstract

This paper analyzes the technical assistance program for research and fishery development, implemented by the Food and Agriculture Organization (FAO) in Brazil, between 1955 and 1978. We argue what were the motivations of the developed countries, the FAO and Brazil to mobilize this knowledge and how the socio-institutional support for its achievement was built. Following the itinerary of experts and attending to the characteristics of the field of fishing biology, we show how the Brazilian field of research, policies and fishing activity were built simultaneously. For this purpose, we used reports from several experts from the FAO and Brazilian public bodies.

## Resumen

Este trabajo analiza el programa de asistencia técnica a la investigación y el desarrollo pesquero, implementado por la Food and Agriculture Organization (FAO) en Brasil, entre 1955 y 1978. Nos interrogamos cuáles son las motivaciones de los países desarrollados, de la FAO y de Brasil para movilizar ese conocimiento y cómo se construyó el soporte socio-institucional para su afincamiento. Siguiendo el itinerario de los expertos y atendiendo a las características del campo de la biología pesquera, mostramos cómo se construyeron, de manera simultánea, el campo de investigación, la política y la actividad pesquera brasileñas. Para eso, recurrimos a los informes de varios expertos de la FAO y de organismos públicos brasileños.

Entre las décadas de 1950 y 1970, Brasil implementó políticas orientadas a industrializar la pesca, construir infraestructura pesquera y desarrollar la industria de procesamiento, modificando el perfil artesanal que tenía el sector, para satisfacer la demanda de productos finos (camarones y langostas) de países centrales y abastecer a la creciente clase media de las grandes urbes brasileras (Diegues, 1983; Abdallah, Bacha, 1999; Goularti Filho, 2016). En este artículo, analizamos otro aspecto de ese proceso de desarrollo, que es el que tiene que ver con el rol del conocimiento experto y de los factores, agentes e intereses trasnacionales que contribuyeron a moldear, de forma casi simultánea, la actividad y los conocimientos pesqueros.^1^ Esa mirada trasnacional es casi ineludible si tenemos en cuenta que, desde la década de 1950, la Food and Agriculture Organization (FAO) puso en marcha programas de asistencia técnica para la pesca en América Latina, que involucraban la formación de recursos humanos en biología pesquera, la provisión de asesoramiento a los gobiernos y la asignación de financiamiento para la investigación y el desarrollo. En línea con esa política, entre mediados de la década de 1950 y mediados de la década de 1960, la FAO financió la visita a Brasil de seis expertos en biología pesquera a través del Expanded Program of Technical Assistance (Epta) de las Naciones Unidas. El asesoramiento de estos expertos, concentrado en la pesca marítima, sentó las bases para la institucionalización de dicho campo y fue el punto de partida para el diseño del Programa de Pesquisa e Desenvolvimento Pesqueiro do Brasil (PDP), de 1967 a 1978, financiado por el United Nations Special Fund (UNSF) (FAO/UN, 1966).

El estudio de este proyecto de asistencia técnica, desde el envío de los primeros expertos en 1955 hasta la finalización del PDP, en 1978, puede contribuir a echar luz sobre algunos aspectos vinculados a la circulación trasnacional de conocimientos entre centros y periferias.^2^ En un plano más bien general lo que nos interrogamos es ¿por qué y cómo determinados conocimientos producidos en instituciones centrales circulan y se institucionalizan en países periféricos? La pregunta no es nueva y fue ampliamente discutida en la historiografía de la ciencia, pero las respuestas no han agotado el tema.^3^ De hecho, el interrogante admite múltiples respuestas en función de diversos factores, como el tipo de conocimiento o campo de investigación, el escenario geopolítico, la intervención o no de intereses y actores extra-científicos y el grado de organización de las relaciones tecnocientíficas trasnacionales. Por ejemplo, en lo que respecta a las motivaciones o intereses, una teoría científica podría circular a través de canales informales, sin ningún tipo de organización *ad hoc* de las relaciones científicas trasnacionales, por el simple interés que despierta en la comunidad académica su capacidad o potencial de responder a problemas de orden cognitivo, que no han encontrado una explicación satisfactoria aún. Sin embargo, dado que la biología pesquera es un campo directamente ligado a la actividad productiva y al rol regulador del Estado (Hubbard, 2014), su circulación e institucionalización en nuevos contextos responde a motivaciones extracientíficas de diversos actores centrales y periféricos.

En ese sentido, Finley (2011, p.8-9) afirma que, en la posguerra, la difusión internacional, a través de la FAO, del concepto de “máximo rendimiento sustentable” (MRS), como marco teórico, regulatorio y legal para la investigación y la actividad pesquera, es “una historia del imperialismo y de la lucha de EEUU, Japón y Europa por mantener el acceso a los *stocks* de peces del hemisferio Sur”.^4^ En este artículo, retomamos estos aportes y los complejizamos a partir de tres preguntas: ¿por qué se consideraba necesario generar capacidades tecnocientíficas vinculadas con la pesca en un país semiperiférico como Brasil? ¿cuáles fueron las motivaciones de la FAO y de los actores de la periferia para movilizar ese conocimiento? y ¿cómo se construyó el soporte socio-institucional y tecnológico a través del cual circuló y se estabilizó ese conocimiento en Brasil? Las dos primeras cuestiones las abordaremos en la primera sección. Allí ofrecemos un panorama de la geopolítica de los recursos y los conocimientos pesqueros, en el que mostramos el interés de las grandes potencias en el mar brasileño e identificamos las motivaciones de dichos países, de la FAO y de diversos actores brasileros para generar capacidades de investigación vinculadas con la pesca marítima en Brasil.

En relación con la tercera pregunta, hay dos aspectos que raramente se han considerado en los estudios históricos sobre la circulación de conocimiento y que pueden ser útiles para pensar tipologías: por un lado, las características de los campos científicos y, por otro lado, la organización de las relaciones tecnocientíficas trasnacionales. Nuestro trabajo pone en foco el carácter aplicado (similar a otros conocimientos involucrados en programas de asistencia técnica) de la biología pesquera, a la que recurre tanto el Estado (para establecer regulaciones restrictivas o mecanismos de promoción) como los empresarios pesqueros (por ejemplo, para saber dónde pescar). En términos generales, podemos decir que es un conocimiento que surge de los requerimientos del modelo industrial de desarrollo pesquero y está metodológicamente ligado a la estadística pública. A su vez, la asistencia técnica es un modo de organización de las relaciones tecnocientíficas trasnacionales que involucra no solo a la comunidad académica, sino también a los Estados, sus burocracias técnicas y, en ocasiones, a los empresarios. En consecuencia, para comprender el rol de los expertos de la FAO en la institucionalización de este campo, no es suficiente observar la “difusión” de una fórmula (como el MRS) entre colegas del centro y la periferia, sino que, además, es necesario registrar sus intervenciones en los procesos de organización institucional y estandarización tecnocognitiva a través de las fronteras entre la academia, el Estado y las empresas. Siguiendo el itinerario de los expertos, en la segunda sección abordamos el proceso de institucionalización de la biología pesquera en Brasil, los mecanismos de coordinación sociocognitiva entre diversos actores y la articulación entre las agendas de investigación y las políticas de desarrollo pesquero.

Nuestro punto de partida para la formulación del problema y el trabajo empírico fue la revisión de los siguientes materiales: (1) literatura sobre la historia de la biología pesquera y de la FAO; (2) bibliografía sobre las políticas pesqueras, el sector pesquero y los antecedentes del campo de la biología pesquera en Brasil; (3) informes de los expertos de la FAO en Brasil (solicitados al archivo del organismo) y documentos del PDP (disponibles en formato digital). En base a esa primera aproximación, hemos relevado otros documentos que nos permitieron completar la reconstrucción histórica.

## Del hemisferio Norte al hemisferio Sur: en busca de nuevos recursos pesqueros

### La geopolítica de los recursos pesqueros y la circulación internacional de expertos

Desde la década de 1940, un sector de la élite científica y técnica brasileña había manifestado interés en el campo de la biología pesquera y puesto en marcha algunos proyectos de investigación (volveremos sobre esto más adelante). Sin embargo, no fue sino hasta mediados de la década de 1950 que comenzó a institucionalizarse ese conocimiento, con la colaboración de expertos de la FAO. Los intereses económicos de las grandes potencias son, desde luego, un factor relevante (entre otros) para explicar la llegada y afincamiento de este campo en las costas brasileñas.

El primer informe enviado a la FAO por el Comité Técnico de Pesca, en 1945, indicaba que muchos recursos del hemisferio Norte (que producía el 93% de las capturas mundiales) habían sido sobreexplotados, mientras que los recursos del hemisferio Sur permanecían subutilizados (Garcia, 1992, p.388). Desde la década de 1930 y, sobre todo, luego de la Segunda Guerra Mundial, el desarrollo de buques factoría, de métodos de conservación a bordo, de dispositivos de localización (ecosondas) y de nuevas artes de pesca condujeron a una intensificación en la explotación de los recursos y a un avance de la pesca de ultramar (Sahrhage, Lundbeck, 2012). Fue en este marco que llegaron los primeros expertos de la FAO a Brasil, coincidiendo con el creciente interés que venían expresando EEUU, Europa y Japón en ciertos recursos pesqueros brasileños.

Desde la Segunda Guerra Mundial y en la inmediata posguerra, el Fish and Wildlife Service (FWS) del Departamento del Interior de los EEUU implementó “proyectos colaborativos científicos y técnicos” con diversos países de América Central. El objetivo de los proyectos fue explorar los recursos pesqueros y asesorar a los gobiernos en su investigación, gestión y explotación (US Department..., 1948). Entre 1955 y 1956, el agregado regional de pesca de la embajada de EEUU en México realizó un estudio para el FWS sobre el potencial de la pesca de camarones en América Latina, en rápida expansión desde el final de la guerra,^5^ para proveer de ese crustáceo al mercado estadounidense. Según el estudio, había una disminución significativa de las capturas de camarón en la costa oeste mexicana, mientras que, en otros países, como Brasil (el segundo productor luego de México), se estimaba que las diez toneladas capturadas en 1955 podían elevarse a 27 en los años siguientes (Lindner, 1957). Entre 1957 y 1958, el Bureau of Commercial Fisheries de EEUU realizó una exploración a bordo del Oregon para estimar el *stock* de camarones en Guyana, Surinam y el norte de Brasil (Bullis, Thompson, 1959). Para 1960, unos cuarenta barcos estadounidenses habían comenzado a pescar camarones frente a las costas de Guyana y a avanzar sobre la costa norte de Brasil (Donini, 1973). Paralelamente, la FAO envió a Brasil al estadounidense John Wise, del Bureau of Commercial Fisheries, para asesorar al gobierno en la conducción de un programa de investigación sobre recursos camaroneros. No obstante, su base de operaciones se concentró en Santos, y, en junio de 1961, el programa lo asumió otro experto de la FAO, el inglés Michael Mistakidis, que también colaboró con los estados de Santa Catarina y Río Grande del Sur. Mientras tanto, en 1963, el Oregon llevó a cabo otra exploración de camarones en el nordeste de Brasil (Wise, 1964).

A su vez, entre 1950 y 1954, la caída de los desembarcos de sardinas en California activó las alarmas del gobierno estadounidense (Clothier, Greenhood, 1956). Desde la década de 1920, el California Department of Fish and Game llevaba a cabo un intenso programa de estudio sobre la sardina del Pacífico, pues era evidente que los *stocks* de esta especie sufrían violentas fluctuaciones, que afectaban a la industria de enlatados (Saic, 1945). Coincidentemente, uno de los dos expertos de la FAO que llegó a Brasil en 1955 fue el estadounidense William E. Ripley, del California Department of Fish and Game (Paiva, 1996). Su trabajo se enfocó en los estados del centro y sur de Brasil (Río de Janeiro, Sao Paulo y Río Grande del Sur), donde se concentraba la captura y procesamiento de sardinas. Según el informe de su sucesor, el noruego Finn Devold (1958, p.25), algunos brasileños afirmaban que Ripley había estimado que el *stock* de sardinas era el más grande del mundo y había posibilidades de capturar hasta quinientas mil toneladas anuales.

También, hacia fines de la década de 1940, algunos países europeos, Japón y EEUU comenzaron a explorar los *stocks* de atunes del Atlántico tropical (Shomura, 1966). Entre 1948 y 1954, Francia realizó una serie de campañas exploratorias y experimentales en la costa de Senegal que fueron seguidas por la llegada masiva de empresas pesqueras francesas y españolas (Dias, Guillotreau, 2004). En ese marco, la costa norte de Brasil se presentaba como un terreno interesante para el desarrollo de nuevas pesquerías atuneras. En 1955, Japón, una vez levantadas las restricciones que le impuso EEUU al finalizar la guerra, comenzó a expandirse en busca de nuevos recursos. Ese año, el buque Toko Maru, de la Agencia Japonesa de Pesca, llevó a cabo una exploración en la costa norte de Brasil con vistas a desarrollar la pesca de atún. También en 1955, llegaba a Brasil, en calidad de experto de la FAO, Robert E.K.D. Lee, instructor de pesca del Bureau of Commercial Fisheries de Hawái. Su misión, realizada entre febrero de 1955 y enero de 1957, se concentró en la recolección de información sobre la pesca de atún y sobre la infraestructura y las prácticas vinculadas a la actividad pesquera, además de asesorar sobre las diversas artes de pesca (Lee, 1957). Para 1961, una docena de palangreros japoneses pescaban atún en las costas de Recife y otros tres en Santos (Wise, Le Guen, 1966). La presencia de compañías japonesas era fruto de un acuerdo con el gobierno de Brasil, según el cual las empresas formarían técnicos locales en el uso de equipamiento de pesca moderno y, al cabo de dos años de operaciones, registrarían sus embarcaciones en el país, incorporando tripulación brasileña (US Fish..., 1959).

Por último, durante la década de 1950, Francia había avanzado en la explotación de langosta en la costa occidental africana (Senegal, Guinea y Mauritania) y, en 1961, comenzó a avanzar sobre el nordeste de Brasil. Dado que Brasil había comenzado a exportar langostas en 1955, la llegada de barcos franceses derivó en un conflicto diplomático (a punto de tornarse militar), conocido como “la guerra de la langosta”. Resuelto el conflicto, varias empresas extranjeras hicieron acuerdos con empresas brasileñas y comenzaron a capturar langostas en las costas de Ceará y Pernambuco (Paiva, 1967).

Desde luego, el avance hacia el hemisferio Sur no se produjo sin conflictos. Desde 1945 y siguiendo la Declaración de Truman que establecía zonas de conservación en las costas de EEUU, diversos países de América Latina reclamaron su soberanía marítima, debilitando el clásico régimen de “libertad de los mares” que prevalecía desde el siglo XIX. El reclamo de México, en 1945, fue seguido por Argentina, en 1946, Chile y Perú, en 1947, y Costa Rica, en 1949 (Finley, 2011).

Ante la escalada de las tensiones, que también afloraron en el hemisferio Norte, en 1952, la International Law Commission redactó un informe en el que recomendaba que la FAO estableciera un marco regulatorio para la protección de los recursos marinos. La International Technical Conference on the Conservation of the Living Resources of the Sea tendría lugar en 1955, en la sede de la FAO. Los meses previos a la conferencia estuvieron atravesados por intensas negociaciones de EEUU con Europa para evitar discutir los reclamos de soberanía y, en cambio, instalar dos tópicos: por un lado, el concepto de MRS como criterio científico para regular la pesca; por otro lado, el principio de abstención, según el cual, cuando un país había realizado estudios científicos sobre el *stock* de una especie, el resto de los estados debían renunciar voluntariamente al derecho de participar en su explotación (Finley, 2011, p.157). Aunque este último principio no recibió el respaldo necesario, EEUU sí logró instalar el MRS no solo como concepto científico, sino también como parte de una doctrina de gestión que aplazaba las restricciones hasta que los modelos matemáticos y las estadísticas probaran los riesgos de continuar pescando. Las puertas estaban abiertas para expandir la pesca sobre territorios distantes, pero también para que las naciones del hemisferio Sur construyeran capacidades con miras a la exploración, explotación y gestión de sus recursos. En ese marco, arribaron a Brasil los primeros expertos de la FAO, provenientes de las principales potencias pesqueras (EEUU, Japón, Finlandia, Gran Bretaña).

### El rol de los organismos internacionales y las políticas públicas brasileras en el desarrollo de las investigaciones sobre recursos pesqueros

Desde la perspectiva de las grandes potencias pesqueras, el envío de expertos y la generación de capacidades de investigación eran cruciales porque permitían obtener información acerca de los recursos pesqueros en otras latitudes. Sin embargo, la historiografía reciente sobre la FAO y otros organismos técnicos internacionales pone de relieve que esos espacios estuvieron atravesados tanto por intereses (y presiones) de las grandes potencias como por ideales humanitarios (Speich Chassé, 2014; Staples, 2006; Jachertz, 2014; Pernet, Ribi Forclaz, 2019). En efecto, en la posguerra, una vez reconstruida Europa y con el avance de los movimientos de descolonización y las tensiones de la Guerra Fría, la cuestión del desarrollo, entendida como la adecuada nutrición de la población vulnerable (en especial con la proteína que proveía el pescado) o como desarrollo de capacidades productivas, se instaló en la agenda de organismos internacionales, de EEUU y de los propios países latinoamericanos que demandaban asistencia financiera y técnica para concretar sus planes. La percepción de ventajas mutuas es, sin dudas, una condición *sine qua non* para la asistencia técnica o la cooperación científica.

A mediados de la década de 1950, cuando arribaron los expertos de la FAO, la cuestión del desarrollo pesquero ya estaba instalada en la agenda de los gobiernos brasileños. En 1934, durante el gobierno de Vargas (1930-1945), se había creado el Departamento de Producción Animal dentro del Ministerio de Agricultura, con un Servicio de Caza y Pesca, encargado (entre otras actividades) de la supervisión de la pesca comercial que se basaría en el Código de Pesca aprobado en 1938. Ese mismo año se puso en marcha un plan para construir mercados de pesca en los centros de mayor producción y consumo. En 1939, la institución filantrópica Abrigo de Cristo Redentor fundó, con apoyo del gobierno, la Escuela de Pesca Darcy Vargas, transferida poco después al Estado Federal para brindar formación técnica y profesional a los pescadores (Caminha, 2019). En 1945, se creó la Caja de Crédito de la Pesca para financiar la compra de equipamientos e insumos y la instalación de pequeñas industrias con fondos recaudados a través de un impuesto del 3% a las capturas que ingresaban en el mercado (US Department..., 1949).

Desde entonces, la búsqueda de asesoramiento experto fue una constante que atravesó a los sucesivos gobiernos. En 1948, la Comisión Técnica Conjunta Brasil-Estados Unidos, constituida a partir de la solicitud de asistencia técnica del presidente Gaspar Dutra, elaboró un informe sobre los factores que promovían o retrasaban el desarrollo económico del país, con una sección especial referida al sector pesquero (US Department..., 1949). En 1952, poco después de que se estableciera la oficina regional de la FAO en Río de Janeiro, el director de la División de Caza y Pesca del Ministerio de Agricultura de Brasil solicitó la asistencia del organismo para evaluar los recursos pesqueros (Ripley, 1956).

El desarrollo de la actividad pesquera también fue apoyado por el gobierno de Juscelino Kubitschek (1956-1961). Para 1960, se habían instalado 16 depósitos equipados con cámaras frigoríficas y fábricas de hielo, gestionados por la División de Caza y Pesca. Por otro lado, se habían incorporado modernas embarcaciones japonesas, noruegas e italianas a la flota pesquera con financiamiento de la Caja Especial de Pesca (Goularti Filho, 2016). En 1962, un documento afirmaba: “Desde 1958 hubo una ‘revolución’ resultante del aumento de barcos grandes, tanto brasileños como extranjeros que vinieron a aumentar las actividades pesqueras hasta un nivel nunca antes alcanzado” (Conselho Nacional..., 1962, p.12). La consecuencia fue un considerable incremento en las capturas: la producción de pescado pasó de 122.410 toneladas, en 1946, a 220.556, en 1960 (Abdallah, Bacha, 1999, p.11).

El crecimiento del sector condujo a la concepción de nuevos organismos de política y gestión dentro del aparato del Estado. En 1962, para evitar superposición de funciones entre diversos organismos y conferir una mayor jerarquía a las políticas destinadas a la pesca, el gobierno creó la Superintendencia de Desarrollo Pesquero (Sudepe), que absorbió al Consejo Nacional de Pesca (creado en 1961), la Caja Especial de Pesca y la División de Caza y Pesca del Ministerio de Agricultura. Su objetivo fue elaborar el primer Plan Nacional de Desarrollo Pesquero, brindar asistencia técnica y financiera a los emprendimientos pesqueros, realizar estudios sobre los recursos, desarrollar códigos de pesca, coordinar programas colaborativos con instituciones extranjeras y asistir a los pescadores en sus problemas económicos y sociales (Goularti Filho, 2016).

No obstante, fue durante el gobierno militar, instaurado en 1964, que se pusieron en marcha políticas mucho más vigorosas para el desarrollo de la pesca industrial. En 1966, la actividad pesquera fue reconocida como “industria de base”, habilitando al Banco Nacional de Desarrollo Económico a destinar recursos al sector. Un año más tarde, el decreto n.60.401 aprobaba el PDP financiado por la UNSF y ejecutado por la FAO, en colaboración con el Ministerio de Agricultura de Brasil.^6^ Ese mismo año, Brasil estableció el otorgamiento de incentivos fiscales para el desarrollo de las industrias de procesamiento o para el equipamiento de las flotas pesqueras. Los incentivos posibilitaron el incremento de las capturas, pero, sobre todo, el crecimiento de un parque industrial para el procesamiento de pescado, que motivó la ocupación de nuevas áreas de pesca y una creciente modernización de la flota. Con oscilaciones en algunos años, Brasil casi duplicó la producción pesquera: pasó de 326.901 toneladas, en 1965, a 646.214 toneladas, en 1978 (Abdallah, Bacha, 1999, p.11).

En este marco, Brasil también necesitaba adquirir capacidades tecnocientíficas para el desarrollo y la gestión de una actividad que prometía crear nuevas industrias o atraer las divisas necesarias para impulsar otros sectores industriales. Pero existe una motivación adicional que incumbía a países como Brasil y a las grandes potencias pesqueras. La biología pesquera se había organizado alrededor de dos objetivos en tensión: por un lado, el de incrementar el alcance e intensidad de la pesca; por otro lado, el de adquirir conocimientos para una mejor conservación de las pesquerías por razones ecológicas o económicas (Hubbard, 2014). En ese sentido, era necesario que los recién llegados al negocio adquirieran conocimientos e información que permitiera la gestión de áreas de pesca comunes a varios países, como la emergente explotación de atún, langostas y camarones en el Atlántico tropical. De hecho, luego de la Segunda Guerra Mundial, el avance de la pesca de ultramar había llevado a una proliferación de comisiones intergubernamentales (más de veinte en 1967), para fomentar la recolección uniforme de datos y la difusión de estadísticas pesqueras y de datos biológicos, además de acordar regulaciones que garantizaran la sustentabilidad (ecológica o económica) de la pesca. Al menos tres de esas comisiones incluían a Brasil: la Comisión Asesora Regional de Pesca para el Atlántico Sudoccidental (Carpas), creada en 1961 e integrada por la Argentina, Brasil y Uruguay; la International Convention for the Conservation of Atlantic Tunas, creada en 1966; y la Western Central Atlantic Fishery Commission (1973), cuyo estatuto puso especial énfasis en explotación y gestión de camarones. La primera y la última comisión estaban dentro de la órbita de la FAO (Carroz, Roche, 1967; Gonçalves, 2019).

## Entre el asesoramiento experto y la pesca como cuestión pública: nacimiento y desarrollo de la biología pesquera en Brasil

### De los museos a las oficinas de estadísticas: la institucionalización de la biología pesquera en Brasil

El programa de la FAO en Brasil se asentó no solo sobre las políticas pesqueras que lo precedían, sino también sobre las inquietudes de la comunidad académica. La decisión de impulsar la actividad pesquera durante el gobierno de Vargas había sido el marco ideal para la concepción de proyectos de investigación ligados a dicho sector. En 1943, la directora del Museo Nacional, Heloisa Alberto Torres, el ictiólogo de la Universidad de Stanford, George Sprague Myers (por entonces de visita en el Museo) y la División de Caza y Pesca del Ministerio de Agricultura de Brasil diseñaron un programa de investigación para mapear las especies de consumo existentes, recolectar datos con miras a construir un sistema de estadística pesquera y estudiar la biología de diferentes especies. Según Torres y Myers, las investigaciones taxonómicas, estadísticas y biológicas eran claves tanto para el desarrollo de la pesca como para su reglamentación con miras a la conservación. Sin embargo, la ejecución del proyecto sufrió varias dilaciones debido a la falta de personal (en Stanford y en Brasil), a las dificultades en el envío de especímenes y a la inestabilidad institucional. En 1952, la directora del Museo Nacional solicitó asistencia a la FAO, pero, cuando la ayuda arribó, en 1955, Torres ya había abandonado su cargo y se dio por finalizado el proyecto (Sá, 2018).

Aun así, el proyecto dejó algunos saldos positivos. En primer lugar, Myers dictó cursos de sistemática de peces y biología pesquera, a los que concurrieron integrantes de diversas instituciones brasileñas (Sá, 2018). En segundo lugar, motivó los primeros trabajos destinados a unificar, en una misma clasificación, los diversos nombres comunes atribuidos por los pescadores a una especie en diversas localidades (cuyos criterios de clasificación solían ser los precios de mercado o las necesidades de la industria secundaria) y correlacionarlos con los nombres científicos utilizados en la sistemática de peces.^7^ Esta homogeneización terminológica entre usos comerciales y científicos era clave para la elaboración de estadísticas pesqueras.

En tercer lugar, el proyecto impulsó las primeras iniciativas de elaboración de estadísticas pesqueras. En 1944, el Departamento de Producción Animal (DPA) de la Secretaría de Agricultura, Industria y Comercio de Sao Paulo (Saic), que se había sumado a la iniciativa del Museo Nacional, creó la Sección de Fauna Marítima, que realizó el primer esfuerzo de recolección datos para las estadísticas de pesca (Saic, 1945). Esa labor fue continuada en la década de 1950 por el Instituto de Pesca Marítima de Santos, sucesor de la Sección de Fauna Marítima (Braga, 1962).

Por último, en el marco de las discusiones sobre las líneas de trabajo del proyecto, la División de Caza y Pesca propuso un plan que trascendía las capacidades del Museo, pues abarcaba investigaciones oceanográficas (Sá, 2018). En ese marco, en 1946, el DPA creó el Instituto Paulista de Oceanografía (IO), con la misión de definir estrategias para el desarrollo de la pesca. Aunque en 1951 el IO fue incorporado a la Universidad de Sao Paulo, continuó teniendo un vínculo fluido con la Saic hasta 1964 (Varela, 2014).^8^ A su vez, el IO motivó la creación de instituciones análogas en otros Estados: en 1953 se crearon la Sociedade de Estudos Oceanográficos do Rio Grande (Seorg) y el Museo Oceanográfico de Río Grande (Torres, 2011),^9^ y en 1952 se dieron los primeros pasos para la creación del Instituto de Biología Marítima y Oceanografía (IBMO) de la Universidad de Recife, que inició sus actividades en 1958 (IBMO, 1959).

La creación de este tipo de instituciones implicaba un abordaje diferente y novedoso de los peces y la pesca, que rompía con la tradición taxonómica característica de los museos. En 1955, Marta Vannucci, integrante del IO, escribió un artículo en el que se refería a la consolidación, durante los últimos decenios, de una “nueva sistemática”, que enfocaba “la clasificación de los seres vivos como algo más que una mera catalogación de nombres aplicados a grupos de individuos idénticos entre sí … que podrían, por lo tanto, ser distribuidos sin retrasos para esa o aquella gaveta de algún Museo de Historia Natural” (Vannucci, 1957, p.217). La nueva sistemática, por el contrario, consideraba a las especies como poblaciones de individuos que ocupaban un área geográfica continua o discontinua, sujetas a variaciones del medio ambiente. La oceanografía (física, química y biológica) podía, por lo tanto, contribuir a correlacionar el estudio de corrientes y afloramientos con la cantidad de nutrientes minerales y sustancia orgánica de las que se alimentan los peces, proveyendo indicadores de concentración de recursos para el desarrollo de pesquerías (Cepal, 29 mar. 1963).

Aunque los expertos de la FAO destacaron la relevancia de este tipo de estudios, pusieron su atención en métodos más directos para estimar el *stock* de peces. En 1955, Ripley dictó un curso de biología pesquera en Santos y diseñó un programa de estadística pesquera basado en datos de desembarcos, obtenidos a través de formularios que debían completar los pescadores. El programa de Ripley apuntaba a: delimitar las áreas de producción de cada especie; estimar la densidad relativa de las especies en cada área (peso por hora de pesca en cada área); evaluar las fluctuaciones estacionales e interanuales en la densidad; estimar el esfuerzo de pesca (horas y artes de pesca para cada especie y área) (Ripley, 1956; Richardson, Moraes, 1960, p.12; Braga, 1962). Los datos relevados permitirían, por lo tanto, evaluar el *stock* de recursos asequibles, el rendimiento de la pesca, la eficacia de las diversas artes de pesca y, a su vez, ofrecer indicadores de sobrepesca, al correlacionar el esfuerzo de pesca con la magnitud de las capturas a lo largo del tiempo.

Ripley (1956) también propuso la realización de estudios biológicos, que apuntaban a determinar el ciclo vital de las poblaciones de una especie a partir de la medición del largo, peso, sexo, edad, curva de crecimiento, momento de desove etc. Dichos estudios eran la base para la investigación sobre dinámica de poblaciones y permitían establecer los parámetros para las mediciones y clasificaciones que realizaban los servicios bioestadísticos, encargados de registrar frecuencias de largo y edad de los efectivos (un dato muy relevante porque la captura de especímenes demasiado jóvenes podía afectar el *stock*). Los sucesores de Ripley, por lo tanto, continuaron impulsando el desarrollo de la estadística pesquera, analizaron algunos de los datos recolectados, asesoraron en el desarrollo de estudios biológicos y colaboraron en la estandarización de métodos de muestreo (Devold, 1958; Richardson, 1961; Wise, 1964). Aunque este conocimiento era aplicable a los recursos pesqueros en general, los expertos colaboraron especialmente con informes y publicaciones referidas a la distribución, abundancia y nivel de explotación de especies de particular interés para las potencias pesqueras, como sardinas, camarones y atunes (Richardson et al., 1960; Neiva, Wise, 1963; Lima, Wise, 1963).

Inicialmente, la principal base de operaciones de los expertos fueron los estados de la región Centro-Sur y, en particular, el estado de Sao Paulo, que era el que contaba con mayores capacidades y recursos para el desarrollo de la biología pesquera. Allí no solo delinearon un programa de trabajo y socializaron a los actores locales en los supuestos teórico-metodológicos y las destrezas prácticas necesarias, sino que también trabajaron en el diseño de arreglos institucionales y en la expansión de la red institucional y cognitiva hacia otros estados. Por ejemplo, durante su estadía (1958-1961), el biólogo inglés Ian Richardson impulsó la colaboración entre el IO y el Instituto de Pesca de Sao Paulo, conformando el Grupo de Investigación sobre Pesca Marítima (conocido como “el Grupo de Santos”) con integrantes de ambas instituciones (FAO/UN, 1966). Asimismo, en abril de 1959, Richardson realizó un viaje, a pedido del Consejo Nacional de Desarrollo Científico y Tecnológico (CNPq), por Salvador, Recife, Fortaleza, San Luis, Natal y Belém y diseñó un programa de investigaciones para la región Norte. Poco más tarde, el IBMO firmó un convenio con la Superintendencia para el Desarrollo del Nordeste (Sudene) que dio origen al Núcleo de Biología Pesquera (IBMO, 1960). Ese mismo año también se fundó la Estación de Biología Marina de la Universidad de Ceará (fundada por Melquíades Pinto Paiva, en Fortaleza). Por último, el informe final de Richardson (1961) consignaba el empeño de Ernesto Tremel, en Santa Catarina, para implementar un programa de investigaciones pesqueras y recomendaba que la Secretaría de Agricultura de dicho Estado apoyara el proyecto proveyendo equipamiento y financiamiento. En 1961, se creó el Departamento Estadual de Caza y Pesca, que albergaría el Centro de Investigaciones de Pesca de Santa Catarina (Paiva, 1996).

Paralelamente, Richardson inició negociaciones con el CNPq y el órgano federal de Caza y Pesca para coordinar los programas de diversos estados, intercambiar sus resultados y estandarizar las mediciones y los métodos utilizados por los diversos grupos regionales. Un paso en esa dirección fue el otorgamiento de becas del CNPq para formar investigadores de diversos estados con el Grupo de Santos (Richardson, 1961). Sus gestiones fueron continuadas por el estadounidense Wise (1961-1964), cuya presencia en Sao Paulo probablemente contribuyó a que el IO recibiera un subsidio de U$ 547.500 de la Fundación Ford, destinado parcialmente a brindar asistencia a empresas privadas y a los departamentos de pesca de los distintos estados (en especial del Nordeste), financiando estadías de formación en biología y tecnología pesquera con el grupo de Santos (Wise, 1964).

En ese contexto, en 1959 comenzaron a organizarse reuniones periódicas de Técnicos de Investigación sobre Pesca Marina, de las que participaban representantes de las diversas instituciones brasileñas antes mencionadas.^10^ A partir de la segunda reunión, se conformaron comisiones especiales para la estandarización de las áreas de pesca, la clasificación de los barcos, los datos recolectados en el mar y durante las descargas, los datos biológicos (especie, longitud, peso etc.) y los métodos y criterios de muestreo. Tanto Richardson como Wise participaron de estas reuniones, asesorando en el diseño y ejecución de programas y proyectos para las diversas instituciones (Wise, 1964). Sobre esa base, y teniendo como trasfondo la reciente creación de la Sudepe, los participantes de la tercera Reunión de Investigadores comenzaron a discutir la elaboración, con asesoramiento de la FAO, de un plan de investigaciones sobre pesca y estudios de tecnología de pescado (Conselho Nacional..., 1962).

Hacia mediados de la década de 1960, ya había varios indicadores de una relativa institucionalización de campo, como publicaciones periódicas y expertos con dedicación exclusiva en Santos, en la Sudene y en el Departamento de Caza y Pesca de Santa Catarina (Wise, 1964). El trabajo de delimitación de las fronteras del campo es también un interesante indicador de institucionalización. En 1965, Marta Vannucci, directora del IO, señaló que existía una gran confusión, incluso entre los propios científicos, respecto de los campos y especialidades que se dedicaban al estudio del mar. Su conferencia versó sobre las diferencias entre oceanografía física, oceanografía química, oceanografía biológica, biología marina y biología pesquera. En su sintético cuadro sobre las ciencias del mar, la biología pesquera ocupaba un lugar especial dentro de la oceanografía biológica, de la que se diferenciaba por su carácter aplicado a resultados inmediatos (Vannucci, 1965). No casualmente, quizás, poco tiempo antes de su conferencia, el IO se había separado del grupo de Santos (Wise, 1964). La biología pesquera adquiría, así, una identidad propia y un objeto mucho más acotado que el de la oceanografía, que debía lidiar con las complejidades de un sistema dinámico e interconectado como el marítimo.

### Nuevas líneas de trabajo y el rol de los expertos de la FAO: desplazamientos entre la ciencia y la política

Como vimos, las características propias de un campo, que suponían desplazamientos entre institutos oceanográficos universitarios y órganos federales o estaduales de caza y pesca, flexibilizaban las fronteras entre ciencia, diplomacia y política. De hecho, hasta 1964, Wise había trabajado, junto con funcionarios de las oficinas regionales de la FAO en Río de Janeiro y en Santiago de Chile, en la elaboración de borradores de un programa de desarrollo pesquero para presentar al UNSF (Wise, 1964). A su vez, el Bureau of Commercial Fisheries de EEUU colaboró con la misión de los expertos del UNSF, llevada a cabo en 1964, para recolectar información y estudiar el modo de mejorar la industria pesquera brasileña. Los expertos realizaron recomendaciones de revisión de leyes, regulaciones, resoluciones y códigos relacionados con la pesca para permitir a los inversores privados operar eficientemente con modernas embarcaciones y equipamiento (US Bureau..., 1966). En 1965, luego de la partida de Wise, Ripley, por entonces jefe de asistencia técnica internacional del Bureau of Commercial Fisheries (1963-1965), regresó a Brasil como experto de la FAO para organizar el PDP, que dirigió entre 1967 y 1969. En ese año, también se desempeñó como asesor de la embajada de EEUU en Brasil (Paiva, 1996).

A diferencia de la asistencia provista por la FAO en el período anterior, el PDP presentaba un plan de operaciones que trascendía los objetivos cognitivos para abarcar aspectos vinculados al modelo de desarrollo pesquero, previendo asesoramiento sobre planificación, arreglos institucionales, regulación, infraestructura, sistema de incentivos al sector privado y formación de funcionarios de gobierno con cargos claves en la administración pesquera. El plan, cuya primera fase duraría dos años, aplazaba para una segunda fase de tres años (sometida a consideración del UNSF) la exploración y evaluación de los recursos pesqueros y la ayuda a instituciones de investigación (Brasil, 11 mar. 1967).^11^

Aunque estaba previsto que el plan de investigaciones se iniciara en 1970, diversas circunstancias aplazaron su implementación hasta 1973. Entre ellas, la falta de personal técnico y la inestabilidad de la Sudepe, que, desde su creación hasta 1973, tuvo 11 superintendentes (SBPC, 1974). Por entonces, los incentivos fiscales habían impulsado la expansión de la pesca, y algunas empresas tenían dificultades para encontrar recursos. Las empresas comenzaron a demandar investigaciones que respaldaran sus emprendimientos, al tiempo que los investigadores reclamaban a la Sudepe que asumiera su rol de orientadora, coordinadora y financiadora de investigaciones de aplicación inmediata. En ese marco, en 1973, comenzó a constituirse un Fondo de Pesca para investigación a partir de contribuciones obligatorias de las empresas (SBPC, 1974).

La falta de equipamiento fue otro de los obstáculos que demoró el inicio del plan de investigaciones. En tanto las estimaciones de *stocks* basadas en los desembarcos de la flota comercial solo registraban las áreas de pesca y no las áreas inexploradas, todos los expertos de la FAO durante el período anterior habían señalado que era necesario disponer de buques de investigación debidamente equipados (Wise, 1964). El acuerdo entre la FAO y el gobierno brasileño para la implementación del PDP fue que Brasil facilitase los barcos de investigación y financiara los cruceros y que la FAO proveyese asesores para el planeamiento y la ejecución de las expediciones pesqueras (Yesaki, 1973). Sin embargo, la adquisición de barcos debidamente equipados se demoró hasta 1973.

A diferencia de las investigaciones realizadas en el período previo, las de este período incorporaron la pesca exploratoria (para la realización de muestreos bio-estadísticos que permitieran identificar nuevas pesquerías), los reconocimientos ecoicos (que permitían calcular la concentración de peces en determinadas áreas) y la pesca experimental (que apuntaba a determinar el poder de captura de un arte de pesca en comparación con otros comúnmente utilizados) (Cepal, 29 mar. 1963). Mitsuo Yesaki (1973), experto de la División de Recursos Pesqueros y Medio Ambiente de la FAO, se encargó de instruir sobre cómo debían organizarse las investigaciones a bordo de los buques de investigación. Yesaki observó que los levantamientos realizados hasta 1973, sobre todo en la costa Sur, no habían seguido un plan integral de trabajo y se habían realizado con embarcaciones obsoletas. Ante este diagnóstico, presentó recomendaciones para intensificar y ampliar las áreas de los levantamientos, lo que permitiría conocer qué recursos había y cuáles eran sus RMSs, y modernizar la flota de investigación con equipamiento para arrastre de popa, redes sintéticas y ecosondas, tecnologías consolidadas a nivel mundial desde la década de 1950, tanto en la flota comercial como en la de investigación (Sahrhage, Lundbeck, 2012). Paralelamente, la FAO puso a disposición el Lamatra y el Cruz del Sur, el último comprado por el PDP de Argentina, para investigar los recursos brasileños. En 1973, se incorporó el Diadorim, que recibió el equipo acústico del Cruz del Sur (PDP, 1975). A lo largo de la década de 1970, el trabajo de los buques de investigación permitió realizar pesca exploratoria sobre la diversidad de regiones y recursos pesqueros brasileños (crustáceos, peces pelágicos y demersales, tiburones etc.), con una amplia variedad de artes de pesca (líneas largas, redes de arrastre de profundidad, medias aguas, cercos, covos etc.), con el fin de buscar nuevos recursos y mejores instrumentos de captura (Haimovici, 2007, p.25-36).

Respecto del desarrollo de investigaciones orientadas a mejorar los rendimientos de las pesquerías, el estado de Santa Catarina, líder en la pesca de camarones y sardinas (Abdallah, Bacha, 1999), fue uno de los más activos. Con el fin de actualizar las técnicas y artes de pesca del sector pesquero, firmó acuerdos de cooperación con la Superintendencia de Desarrollo del Sur y el Banco Regional de Desarrollo del Extremo Sur. Los acuerdos incluyeron el desarrollo de investigaciones, que contaron con el asesoramiento de David Lintern, de la FAO, y Ernesto Tremel, de la Sudepe y del Centro de Investigaciones de Pesca de Santa Catarina. Estos investigadores organizaron equipos (compuestos por un economista, un contador y un ingeniero especializado en barcos), para realizar un relevamiento sobre las flotas pesqueras (sardineras y camaroneras) y establecer cuáles hacían mejor provecho de los recursos, tanto en términos de ganancias como de conservación. Las empresas pesqueras participaron de las investigaciones, en particular las más grandes, ya que contaban con datos sobre los recursos capturados, los rendimientos de pesca y aspectos financieros. En las conclusiones de las investigaciones, los expertos plantearon la necesidad de renovar las embarcaciones, incorporar ecosondas para detectar los cardúmenes, utilizar redes más selectivas y artes de pesca modernas (Superintendência..., 1972).

Paralelamente, en 1974, con el fin de formar investigadores sobre problemas pesqueros que pudiesen asesorar a la Sudepe, el PDP creó el Grupo de Trabajo y Entrenamiento sobre Evaluación de *Stocks* (GTT), que se reunió por primera vez en el Instituto de Pesca de Santos. El GTT organizó diez grupos, cada uno para investigar un recurso pesquero de importancia comercial. Las principales instituciones brasileñas enviaron investigadores a los cursos del GTT sobre modelos de evaluación de recursos y fórmulas para el cálculo del MRS, dictados por L.K. Boerema, de la División de Recursos Pesqueros y Medio Ambiente de la FAO (PDP, 1974).

El GTT permitió la formación inicial de 42 investigadores, biólogos principalmente. En los años subsiguientes, el GTT continuaría funcionado mediante reuniones periódicas para definir el estado de explotación y conservación de los recursos. Asimismo, organizó grupos de estudios permanentes especializados en los recursos más explotados: sardinas, camarones, langostas y piramatuba, el primero destinado al consumo de las clases medias brasileras y los últimos tres al mercado estadounidense (PDP, 1981). El objetivo de estos grupos fue hacer un seguimiento permanente del MSR de los recursos pesqueros y comunicárlo a la Sudepe para que sus funcionarios desarrollasen regulaciones públicas, tales como volúmenes máximos desembarcados y tamaños mínimos en las mallas de las redes de pesca.^12^ Varios de los líderes de los grupos permanentes, como Ernesto Tremel, Gelso Vazzoler, Getulio de Souza Neiva y Soloncy José Cordeiro de Moura, habían participado de las investigaciones que emprendieron los primeros expertos de la FAO en el período anterior (Paiva, 1996). En algunos casos, las empresas pesqueras participaban de las reuniones de los grupos permanentes de estudios. Un rol destacado lo tuvo la Compañía de Conservas Coqueiro, la más importante del país, que estableció su propio sistema de recolección de datos para la pesca de sardina y presentó informes, producidos a partir de entrevistas a bordo. La semejanza entre los datos recolectados por Coqueiro y por los expertos del PDP permitió evaluar el estado de explotación de las sardinas y proponer medidas para su mejor aprovechamiento (PDP, 1977a).

Finalmente, las estadísticas pesqueras, iniciadas con el programa de Ripley en 1955, se desarrollaron gracias a la creación de la Unidad de Planeamiento y Colecta de Datos Básicos, con sede en Brasilia, que se encargaría de formar funcionarios para poder realizar encuestas a los pescadores. En 1973, esta unidad desarrolló el Sistema de Mapas de a Bordo que utilizarían los encuestadores de las diversas bases de operaciones establecidas por el PDP en los principales estados pesqueros brasileros (Río de Janeiro, Santa Catarina, Río Grande del Sur, Río Grande del Norte, Paraíba y Ceará) (PDP, 1977b). Las nuevas estadísticas producidas por el PDP recopilaron mayor diversidad de datos, como características técnicas de los barcos, latitudes y batimetrías de las zonas pesca, cantidad de lances efectuados, días de pesca, días de navegación, fauna acompañante y características biológicas del pescado. La Unidad no solo se encargó de producir datos nuevos, sino también de recopilar, homogeneizar y sistematizar los datos ofrecidos por la Sudepe, las colonias de pescadores, las empresas, el Ministerio de Agricultura y la Cartera de Comercio Exterior del Banco de Brasil (PDP, 1974). Para finales de la década de 1970, Brasil contaba con un exhaustivo “inventario básico” sobre el complejo pesquero, que recopilaba datos sobre flotas, capturas, industria y comercio (PDP, 1981).

## Consideraciones finales

El interés de países como Japón, EEUU y Francia en los recursos pesqueros brasileños fue el telón de fondo sobre el que se desplegó la asistencia técnica. De hecho, la circulación de expertos desde instituciones centrales a periféricas puede utilizarse como un canal diplomático para interactuar no solo con científicos locales, sino también con sus autoridades políticas y burocracias técnicas. En ese sentido, mostramos que los expertos pueden cumplir el rol de “agentes de inteligencia de recursos” o de diplomáticos, en tanto se encargan de producir información sobre los recursos claves y de asesorar en el diseño de programas de desarrollo. Ciertamente, es factible que los expertos no siempre desempeñen ese rol y, de hecho, aquí solo hemos podido mostrarlo con más claridad con relación a los expertos de EEUU.^13^

Ahora bien, sería un poco simplista atribuir la asistencia técnica de la FAO, exclusivamente, a los intereses de las grandes potencias. En primer lugar, porque la FAO, e incluso EEUU, tenía una genuina preocupación por el desarrollo, sea por razones humanitarias o bien para neutralizar cualquier atisbo de movilización social y afinidad con el comunismo (Buckley, 2021). En segundo lugar, porque la FAO, junto a otros organismos regionales, buscó generar conocimientos y normas para la gestión regional e internacional de las explotaciones pesqueras, que a menudo trascendían las fronteras de los estados nacionales. En tercer lugar, porque los propios países latinoamericanos aprovecharon esa preocupación para hacer avanzar sus propias agendas de desarrollo e investigación. En efecto, desde la década de 1940, el gobierno y los científicos brasileños buscaron asesoramiento y asistencia, tanto en EEUU como en la FAO, para mejorar su performance en la actividad pesquera. Las políticas pesqueras implementadas en Brasil, e incluso en otros países de América Latina (como Chile y Perú), ponen de relieve que los gobiernos de la región fueron muy activos en la promoción del modelo industrial de desarrollo pesquero (Thorpe, Ibarra, Reid, 2000).

De hecho, en el programa desplegado en colaboración con los expertos de la FAO en Brasil, hemos identificado dos etapas en las que las agendas de investigación se correlacionaron con las políticas para el sector. En la primera etapa, que transcurrió entre 1955 y 1965, los expertos definieron parámetros para la recolección de datos estadísticos y sentaron las bases para el estudio biológico de las especies de interés comercial. La definición de una agenda de investigación más centrada en la cuestión de la pesca como actividad productiva, que en los mecanismos de bio-producción del océano, institucionalizó parcialmente un nuevo campo de conocimiento más ligado al problema del desarrollo económico. En la segunda etapa, que coincidió con las políticas pesqueras orientadas a expandir e intensificar la pesca, la asistencia de la FAO se concentró en la exploración de nuevas áreas y artes de pesca (a veces en colaboración con empresarios pesqueros), en la transmisión de conocimientos para el estudio de la dinámica de poblaciones y el cálculo de los MRSs.

Por último, nuestro trabajo puso de relieve que la circulación de conocimientos entre instituciones centrales y periféricas y su efectiva institucionalización involucra procesos mucho más complejos que la transferencia de fórmulas matemáticas (como los MRS), en especial en campos como la biología pesquera, cuya dinámica atraviesa las fronteras entre el sector productivo, las instituciones científicas y los organismos públicos (Hubbard, 2014). En efecto, la circulación de conocimientos expertos supuso el despliegue de un suporte socio-institucional que articuló la participación de estos diferentes actores. Este soporte se construyó a partir de dos procesos. El primero supuso el desarrollo de un programa de investigación y formación de nuevos investigadores, que coordinó los criterios de investigación de las diversas instituciones del litoral brasilero. El segundo, estrechamente vinculado al punto previo, supuso procesos de estandarización tecno-cognitiva entre científicos, burocracias técnicas y empresas pesqueras. Algunos ejemplos de ese proceso fueron: la búsqueda de un sistema de clasificación común para científicos, técnicos y pescadores; la difusión de artes de pesca (como las redes sintéticas) e instrumentos (como los ecosondas) comunes a la pesca experimental, exploratoria y comercial (que permitía hacer investigaciones a bordo de buques de investigación o comerciales); y la unificación de criterios de medición (áreas de pesca, batimetrías, esfuerzo de pesca, tamaño y edad de los peces, etcétera) entre investigadores, técnicos de los servicios estadísticos y pescadores que completaban formularios. A su vez, la creación de la Sudepe, que con el tiempo asumió cierto liderazgo en la homogeneización de las estadísticas y constituyó grupos de trabajo por especie, pone de relieve que la asistencia técnica implicó reformas institucionales que permitieron materializar en el Estado brasilero los núcleos conceptuales organizadores del campo y la actividad pesquera.

## NOTAS

1 En este sentido, nuestro trabajo puede ser un aporte a la literatura reciente sobre el rol de los expertos en la materialización de los ideales de desarrollo y modernización forjados en el contexto de la Guerra Fría (Lorek, Chastain, 2020).

2 Aunque el núcleo temporal de nuestra investigación es el período 1955-1978, en las siguientes secciones también reseñamos antecedentes útiles para identificar cambios y continuidades en los ejes de análisis, o bien para mostrar el escenario sobre el que se asentó el programa de la FAO.

3 La literatura sobre el tema es muy extensa; por razones de espacio, remitimos a dos autores de referencia ineludible como Secord (2004) y Krige (2019).

4 El concepto fue desarrollado por el científico y asesor pesquero estadounidense, Milner Schaefer y establecía cuánto se podía pescar de un recurso antes de hacerlo colapsar. En la década de 1960 el cálculo de los MRS devino el núcleo conceptual de la biología pesquera a nivel internacional (Finley, 2011). El trabajo de Martins (2018) fue, quizás, uno de los primeros que hizo referencia a la difusión de este concepto en Brasil a partir de la misión de Ripley, uno de los expertos de la FAO.

5 Como antecedente, debemos mencionar que, al terminar la guerra, EEUU exploró el potencial de pesca camaronera en la costa este mexicana, Honduras y Nicaragua (Finley, 2011, p.149).

6 El PDP contó con 420 mil USD del UNSF e igual monto de la contraparte brasilera (Brasil, 11 mar. 1967).

7 Entre fines de la década de 1940 y principios de la siguiente, al menos dos integrantes del proyecto publicaron documentos con los nombres vulgares atribuidos a los peces en los principales puertos de los estados de Río de Janeiro, Pernambuco y Bahía, con sus correspondientes nombres en sistemática. Durante su estadía, Wise realizaría gestiones para darle continuidad a este proyecto (Ripley, 1956; Wise, 1964).

8 Sobre la institucionalización de la oceanografía en Brasil, véase también Lopes (2021).

9 La Seorg impulsó la firma de un convenio con el Departamento Estadual de Puertos, Ríos y Canales, a partir del cual la primera institución realizaría investigaciones oceanográficas en el litoral riograndense y la segunda proveería materiales y embarcaciones para dichas actividades (Torres, 2011).

10 Las primeras reuniones se realizaron en Río de Janeiro (1959), Santos (1961), y Florianópolis (1962) (Richardson, 1961; Wise, 1964).

11 Inicialmente, quienes estuvieron a cargo de las investigaciones del PDP fueron Haroldo Travassos, Soloncy José Cordeiro de Moura (que venía de realizar estadías de formación en EEUU, Japón y la URSS) y el estadounidense Edward C. Greenhood (Paiva, 1996).

12 Estas regulaciones, originalmente desarrolladas por instituciones centrales, caracterizaron el marco regulatorio pesquero latinoamericano desde la década de 1960 (Thorpe, Ibarra, Reid, 2000).

13 En efecto, el trabajo de Dinius (2019) sobre los proyectos de asistencia técnica implementados en la década de 1950 por la FAO en el Amazonas brasileño muestra que los expertos no deben considerarse, necesariamente, como agentes del imperialismo.
